# Current Insight into Biological Markers of Depressive Disorder in Children and Adolescents: A Narrative Review

**DOI:** 10.3390/antiox14060699

**Published:** 2025-06-09

**Authors:** Jana Trebatická, Martin Vatrál, Barbora Katrenčíková, Jana Muchová, Zdeňka Ďuračková

**Affiliations:** 1 Department of Paediatric Psychiatry, Faculty of Medicine, The National Institute of Children’s Diseases, Comenius University, Limbová 1, 833 40 Bratislava, Slovakia; jana.trebaticka@fmed.uniba.sk (J.T.); vatral3@uniba.sk (M.V.); 2 Faculty of Medicine, Institute of Medical Chemistry, Biochemistry and Clinical Biochemistry, Comenius University, Sasinkova 2, 813 72 Bratislava, Slovakia; barbora.katrencikova@fmed.uniba.sk (B.K.); jana.muchova@fmed.uniba.sk (J.M.)

**Keywords:** depressive disorder, neurotransmitter, inflammation, neuroendocrine system, oxidative stress, lipid profile

## Abstract

Depressive disorder (DD) in children and adolescents is a growing public health concern with a complex and multifactorial etiology. While most biomarker research has focused on adults, increasing attention is being paid to age-specific molecular mechanisms. This narrative review provides a comprehensive overview of current knowledge on potential biomarkers of DD, including genetic, neurotransmitter, hormonal, inflammatory, lipid, and oxidative stress markers, in youth compared to adult populations. Special emphasis is given to findings from the DEPOXIN project (Molecular basis of depressive disorder in children and adolescents, the influence of omega-3 fatty acids and oxidative stress), a multicenter study investigating biological markers in children and adolescents with DD. The project identified significantly increased oxidative stress markers (8-isoprostanes, advanced oxidation protein products, nitrotyrosine) and decreased antioxidant enzyme activity (glutathione peroxidase). Moreover, HDL (high density lipoproteins) cholesterol and its subfractions were negatively correlated with depression severity. At the same time, thromboxane B2, omega-6/omega-3 fatty acid ratios, and salivary cortisol levels showed strong positive correlations with depressive symptoms and biochemical markers of inflammation. These results suggest a distinct molecular profile of depression in paediatric populations, emphasizing the importance of developmental context in biomarker research. The review aims to synthesize existing evidence, compare findings across age groups, and highlight the need for personalized, age-appropriate strategies in the diagnosis and treatment of depressive disorders.

## 1. Introduction

Depressive disorder (DD) is one of the most common multifactorial psychiatric disorders affecting children and adolescents. It significantly impairs psychosocial development and places a substantial burden on families and society as a whole [1]. In addition to the basic symptoms (such as sadness, fatigue, lack of energy, loss of interest in peers, sleep disorders, eating disorders), pediatric depression is also manifested by poor academic performance, interpersonal relationships, and is closely related to suicidal behavior [2]. It is influenced by genetic, environmental, psychological, and biological factors. While diagnosis is based on clinical symptomatology, objective diagnostic tools remain lacking. Treatment outcomes are often unsatisfactory, with over 60% of patients showing resistance to first-line therapies [3]. Treatment-resistant depression (TRD) in the pediatric population can be defined as the presence of clinically significant depressive symptoms that persist despite an adequate trial of evidence-based psychotherapy and an antidepressant with Grade A evidence for pediatric depression, such as fluoxetine, escitalopram, or sertraline [4]. There is great variability in approaches to TRD across European countries. However, in Italy, a consensus therapeutic strategy for treating TRD was developed using a Delphi panel. A high level of consensus and agreement was reached on the importance of adding lithium and/or antipsychotics as augmentation therapy, as well as on the need for long-term maintenance treatment. A high level of consensus and agreement was also reached on the identification of esketamine nasal spray as the best option for patients with TRD and on the safe administration of esketamine in an outpatient setting [5]. However, Slovak clinicians often refer to international guidelines and adapt them to local clinical practice. Unfortunately, in Slovakia, esketamine and lithium are not approved for use in patients under 18 years of age.

While the prevalence of depressive episodes in adolescents has gradually increased, it varies significantly across regions. The American Academy of Child and Adolescent Psychiatry estimates a lifetime prevalence of major depressive disorder in adolescents at approximately 11%, with higher rates among females than males [6].

In Europe, the Saving and Empowering Young Lives in Europe (SEYLE) project reported a wide range in adolescent depression rates, from 7.1% in Hungary to 19.4% in Israel [7]. A systematic review and meta-analysis estimated the prevalence of depressive disorder among European children and adolescents to be approximately 1.7%. The prevalence was higher among secondary school children (2.5%) than primary school children (0.6%). Additionally, among secondary school children, females were more prevalent than males [8].

In Slovakia, specific data remain scarce. However, a population-based study reported a current depressive disorder prevalence of 2.56%, with higher rates among women (3.24%) than men (1.84%) [9].

Variability in reported prevalence is likely due to differences in study design, diagnostic criteria, cultural context, and access to mental health services.

**Figure 1:**
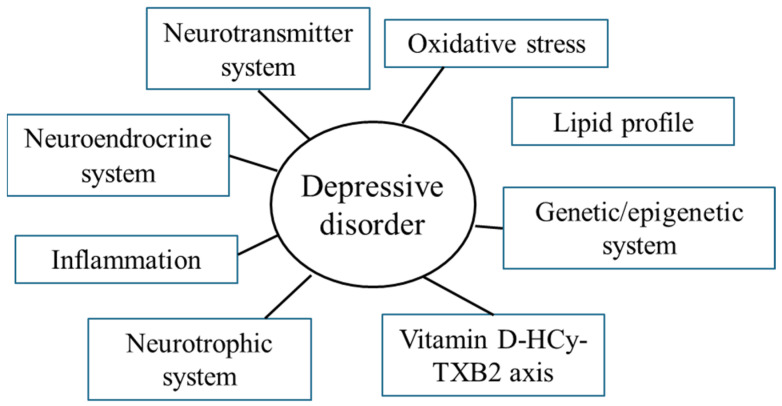
Systems involved in the pathophysiology of depressive disorder. HCy—homocysteine; TXB2—thromboxane B2.

Research on the biological basis of DD has intensified, particularly in adults. However, studies in children and adolescents are comparatively limited. Emerging evidence suggests that specific biological markers, ranging from genetic and epigenetic changes to alterations in neurotransmitters, hormones, immune responses, and oxidative stress, may enhance diagnostic accuracy and therapeutic targeting (Figure 1).

This narrative review integrates findings from previous studies with results from the DEPOXIN project (ISRCTN 81655012), which analyzed various biomarkers in children and adolescents with depression (*n* = 60) compared to healthy controls (*n* = 20). Inclusion criteria included the diagnosis of depressive disorder or mixed anxiety and depressive disorder, age 7–18 years, with normal eating habits and no indication of chronic somatic disease. The diagnoses were determined according to the International Classification of Diseases, 10th edition (ICD-10). Exclusion criteria were chronic somatic diseases (endocrine, metabolic, autoimmune), dietary restrictions (vegetarians, lactose intolerance, celiac disease), psychotic disorders, eating disorders, addiction to psychoactive compounds, personality disorders, organic mental disorders, and pervasive developmental disorders. In the presented work, baseline data (obtained when patients were included in the project) were evaluated. Ratings of depressive severity were made using the self-rated scale of the Children’s Depression Inventory (CDI) [10]. Potential markers from the DEPOXIN project as well as from other publications, are listed in Supplemental Table S1.

Investigated parameters include: the omega-6/omega-3 fatty acid ratio [10], lipid profile and HDL subfractions [11], inflammatory markers (homocysteine, thromboxane), brain-derived neurotrophic factor (BDNF), vitamin D [12], oxidative stress markers [13], stress hormones [14], and tryptophan catabolism metabolites [15]. This review aims to look for potential biomarkers of depressive disorder and point out their mutual relationships in children and adolescents.

Early identification and intervention remain crucial to prevent adverse long-term outcomes that extend into adulthood.

## 2. Genetic and Epigenetic Markers

Recent advances in genomic research highlight the complex, polygenic nature of depression. A global study involving over 5 million individuals from 29 countries—one-quarter of whom were of non-European ancestry—identified 700 genetic variants associated with depression, including 293 previously unknown ones [16].

Among the most studied genetic variants associated with depression is 5-HTTLPR, a polymorphism in the serotonin transporter gene (SLC6A4). This polymorphic region has been linked to depression, especially in youth, where its interaction with environmental stressors appears to elevate risk [17,18,19]. However, this relationship has not been confirmed in the adult population [20].

Epigenetic mechanisms, particularly DNA methylation, also play a critical role in depression pathogenesis. These modifications can alter gene expression in response to environmental stimuli such as stress or trauma [21,22,23].

Emerging research also points to mitochondrial DNA (mtDNA) involvement. One study showed increased methylation of the mtDNA D-loop in patients with DD compared to those with bipolar disorder and healthy controls. Remission was associated with decreased mtDNA copy number and reduced D-loop methylation, suggesting a potential biomarker for DD progression [24,25].

## 3. Neurotransmitters and Their Metabolites

Alterations in neurotransmitter systems are fundamental to the pathophysiology of depression across all age groups. The serotonergic, dopaminergic, and noradrenergic systems are still developing in children, which may affect how depression manifests and responds to treatment compared to adults.

Key neurotransmitters involved include:

- Serotonin (5-hydroxytryptamin, 5-HT): Reduced levels of 5-HT and its metabolite 5-hydroxyindole acetic acid (5-HIAA) have been documented in depressed individuals [26,27]. Direct comparisons of baseline serotonin levels across age groups are limited. The observed differences in serotonin transporter (SERT) expression and selective serotonin reuptake inhibitor (SSRI) efficacy suggest that the serotonin system matures over time and potentially influences the presentation and treatment of depression. These developmental variations underscore the importance of age-specific approaches in diagnosing and treating depressive disorders.
- Noradrenalin (NA) (norepinephrine): NA affects attention, arousal, and stress responses in adults. Deficits in NA are associated with fatigue and concentration difficulties in depression. The noradrenergic system’s development in children may influence how stress and attention-related symptoms present in pediatric depression. Attention is currently being paid to studying a subset of depressed patients who might respond to new selective noradrenalin reuptake inhibitors. Understanding these variations is crucial for developing effective, age-appropriate treatments [28].
- Dopamine (DA): DA influences motivation, pleasure, and motor function. Hypoactivity is observed in DD and contributes to symptoms like anhedonia. The nucleus accumbens (NAc), also known as the ventral striatum, is a key brain region involved in reward, motivation, and decision-making. It plays a crucial role in processing both positive and negative emotions and is implicated in various neurological and psychiatric disorders, including depression. The NAc and its dopaminergic input from the ventral tegmental area (VTA) form the mesolimbic dopamine system in depression. The mesolimbic dopamine system is most commonly associated with the rewarding effects of food, sex, and drugs. Given the importance of anhedonia, reduced motivation, and reduced energy levels in most individuals with depression, it is assumed that the NAc and VTA contribute significantly to the pathophysiology and symptomatology of depression and may even be involved in its etiology. Recent studies show that manipulations of key proteins (e.g., cAMP response element-binding protein (CREB,) dynorphin, brain-derived neurotrophic factor (BDNF)) in the rodent VTA-NAc circuit produce unique behavioral phenotypes, some of which are directly related to depression. Studies of these and other proteins in the mesolimbic dopamine system have created new approaches to modeling key symptoms of depression in animals and could allow the development of antidepressant drugs with fundamentally new mechanisms of action [29].
- Glutamate/GABA: Depression is linked to an imbalance between excitatory (glutamate) and inhibitory (GABA) neurotransmission. The paradigm shift from the monoamine hypothesis of depression to the glutamate-centered neuroplasticity hypothesis may represent a substantial advance in the hypothesis that drives research into new drugs and therapies [30].

**Figure 2:**
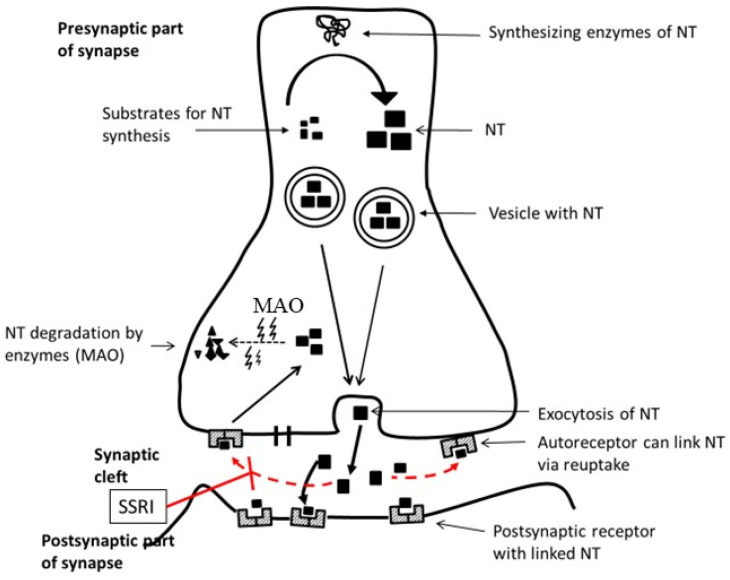
Neurotransmitters in the synapse. NT—neurotransmitter; MAO—monoamine oxidase; SSRI—selective serotonin reuptake inhibitor; 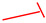
—symbol of inhibition.

Molecules of neurotransmitters (NT) (Table 1) are synthesized from their precursors by enzymes and are stored in vesicles. Vesicles are released into the synapse cleft after the action potential fuses with the presynaptic membrane and NT. Released NT is linked to postsynaptic receptors, and a signal is transferred (→) to the postsynapse. NT can be reuptaken by auto receptors and neurotransmission is inhibited. Reuptake of NT (reduction of, for example, serotonin in the synaptic cleft) is inhibited by SSRI (serotonin is increased in the cleft and neurotransmission is increased). Reuptaken NT can be enzymatically degraded (MAO) (Figure 2).

### 3.1. Developmental Considerations

Children and adolescents exhibit lower serotonin transporter (SERT) expression and function than adults, which may explain reduced responsiveness to SSRIs in younger populations. Browman et al. [31] and Wang et al. [32] found that neurotransmitter dysregulation in adolescents with first-episode DD primarily involves catecholaminergic systems (dopamine and noradrenalin) whereas adults exhibit broader disturbances.

Notably, Gabbay et al. [33] reported reduced GABA levels in the anterior cingulate cortex of adolescents with anhedonic depression, but not in those without anhedonia. While serotonin, dopamine, and noradrenaline are central to the pathophysiology of depression in both children and adults, developmental differences necessitate age-specific approaches to diagnosis and treatment. Understanding these nuances is crucial for effective management of depression across the lifespan.

### 3.2. Summary

Alterations in neurotransmitter systems play a critical role in the pathophysiology of depression, with distinct developmental patterns observed across age groups. While serotonin, noradrenalin, dopamine, and glutamate/GABA systems are all involved, their dysfunction and the corresponding treatment responses vary between children, adolescents, and adults. These age-specific differences underscore the need for customized approaches in both the diagnosis and treatment of depression, particularly as neurotransmitter dysregulation shifts with age. Further research into the developmental aspects of neurotransmitter systems will be essential for optimizing age-appropriate interventions.

## 4. Hormonal and Endocrine Markers

The hypothalamic–pituitary–adrenal (HPA) axis plays a central role in the neuroendocrine regulation of stress. Dysregulation of the HPA axis is a well-established feature of depressive disorders, though its manifestations differ across developmental stages.

**Figure 3:**
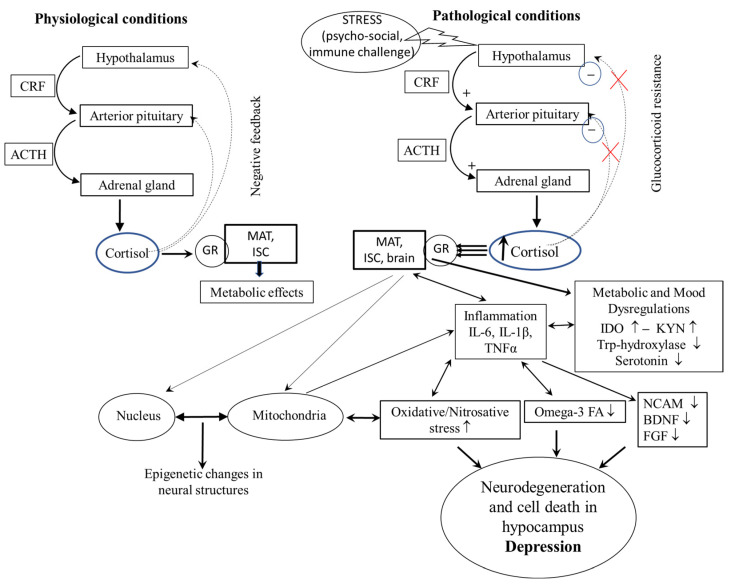
HPA axis and inflammation under physiological conditions and chronic stress. HPA—hypothalamic-pituitary-adrenal axis; CRF—corticotropin-releasing factor; ACTH—adrenocorticotropic hormone; GR—glucocorticoid receptor; IL-6—cytokine IL-6; IL-1β—interleukin-1β; TNF-α—tumor necrosis factor α; ISC—immune system cells; MAT—metabolically active tissue; NCAM—neural cell adhesion molecule; BDNF—brain-derived neurotrophic factor; FGF—fibroblast growth factor; IDO—indoleamine-2,3-dioxygenase; Trp—tryptophan; FA—fatty acids; KYN—kynurenine. The direction of the arrow means that one process/substance will affect another process/tissue. Modified according to [35].

Under normal conditions, cortisol secretion is regulated via negative feedback. Chronic stress disrupts this balance, leading to persistent cortisol elevation and glucocorticoid receptor (GR) resistance [34] (Figure 3). This contributes to neuronal damage through mechanisms such as mitochondrial dysfunction, oxidative stress, and decreased BDNF availability.

**Figure 4:**
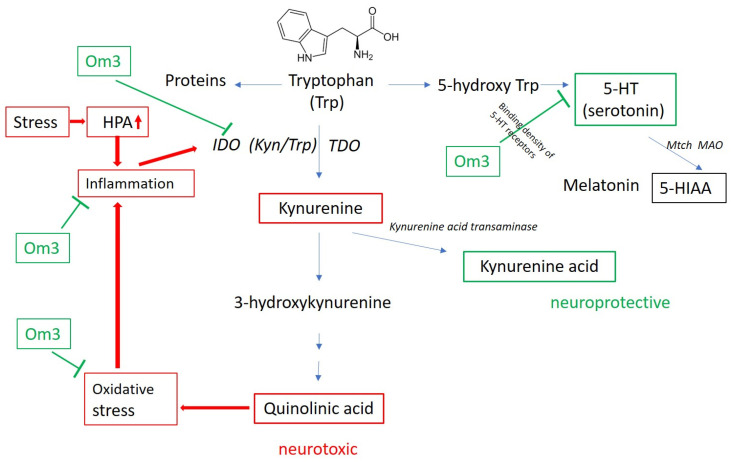
Catabolism of tryptophan (according to [15]). TDO—tryptophan 2,3-dioxygenase; IDO—indoleamine 2,3-dioxygenase; 5-HT—5-hydroxytryptamine; 5-HIAA—5-hydroxyindolacetic acid; Om3—omega-3 fatty acid; HPA hypothalamic-pituitary-adrenal axis; 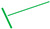
—symbol of inhibition.

Hypercortisolism may contribute to serotonin (SER) depletion through activation of the initiating enzymes tryptophan 2,3-dioxygenase (TDO) and indoleamine 2,3-dioxygenase (IDO), which divert tryptophan (Trp) metabolism toward the kynurenine pathway. In addition to chronic stress, oxidative stress and inflammation are known activators of this pathway. In the brain, serotonin is synthesized from Trp, although only 1–2% of total Trp is converted to serotonin within the central nervous system. Once synthesized, SER is rapidly metabolized by mitochondrial monoamine oxidase (MAO) into its stable end-product, 5-hydroxyindoleacetic acid (5-HIAA) (Figure 4).

Inflammatory signals, often initiated by HPA axis activation in response to stress, trigger the kynurenine pathway, leading to increased production of neuroactive metabolites such as kynurenine and the neurotoxic quinolinic acid (QA). However, kynurenine can also be metabolized into kynurenic acid, which has neuroprotective properties.

In the DEPOXIN project, we observed a significantly elevated kynurenine/tryptophan (KYN/TRP) ratio, along with reduced levels of 5-hydroxytryptophan (5-HTP) and serotonin, and increased 5-HIAA in patients with depressive disorder compared to healthy controls. Furthermore, a positive correlation between KYN/TRP ratio and cortisol levels in depressed adolescents supports the link between HPA axis dysregulation and altered tryptophan metabolism. Omega-3 fatty acids may interfere with the processes of the kynurenine and serotonin pathways, which may have some therapeutic significance [15].

Other hormonal influences, such as subclinical hypothyroidism, have also been noted in some depressed patients [36].

### 4.1. Developmental Variability in Cortisol Reactivity

Cortisol responses vary by age. Children may exhibit blunted responses (hypocortisolism), while adolescents, especially females, show heightened reactivity [37,38]. A meta-analysis by Lopez-Duran et al. [39] confirmed HPA axis hyperactivity in depressed youth, marked by impaired cortisol feedback regulation.

Epigenetic modifications of the NR3C1 gene (glucocorticoid receptor) have been associated with increased depression risk [40,41]. Protein prolyl isomerase 5 (FKBP5), a GR co-chaperone, has also been implicated in depression and suicidal behavior, particularly via single-nucleotide polymorphisms in adults [42,43].

In the DEPOXIN study, baseline salivary cortisol levels did not differ significantly between depressed and healthy youth. However, cortisol levels positively correlated with the severity of depressive symptoms assessed using CDI (Children’s Depression Inventory) scores and oxidative stress markers, suggesting a relationship between cortisol dysregulation and depression severity [14].

### 4.2. Summary

The HPA axis plays a central role in regulating stress and is significantly implicated in depressive disorders, with its dysregulation differing across developmental stages. Chronic stress and hypercortisolism contribute to serotonin depletion and altered tryptophan metabolism, leading to neurotoxic metabolites and neuronal damage. Developmental differences in cortisol reactivity highlight the importance of age-specific factors in depression, with heightened cortisol responses observed in adolescents, particularly females. Additionally, genetic and epigenetic factors influencing glucocorticoid receptor function further complicate the relationship between stress and depression. These findings underscore the need for tailored approaches in understanding and treating depression, particularly in youth, and suggest potential therapeutic interventions targeting the HPA axis and serotonin pathways.

## 5. Immune and Inflammatory Markers

Depressive disorder (DD) has been increasingly associated with dysregulation of the neuroimmune system and chronic inflammation. Evidence from both adult and pediatric populations suggests that depression is linked to alterations in immune function, particularly involving pro-inflammatory cytokines such as interleukin-6 (IL-6), interleukin-1β (IL-1β), tumor necrosis factor-alpha (TNF-α), and interferon-gamma (IFN-γ).

In adults with depression, elevated levels of IL-6 and, to a lesser extent, TNF-α and C-reactive protein (CRP) have been consistently reported [44,45]. These inflammatory markers are believed to contribute to depressive symptoms by influencing neurotransmitter metabolism, neuroendocrine function, and neural plasticity. The “cytokine hypothesis” of depression proposes that chronic inflammation disrupts brain function, thereby maintaining depressive symptoms [46,47,48,49].

Emerging research in pediatric population suggests a similar relationship between inflammation and depression. Studies have found increased levels of pro-inflammatory cytokines and reduced levels of anti-inflammatory cytokines, such as IL-10, in children and adolescents with DD. Notably, adolescents with depression may show heightened activation of immune-related biological pathways, like HPA axis [14], or the kynurenine pathway [15], compared to adults [49]. This suggests that the developing neuroimmune system may be particularly sensitive to inflammatory processes, potentially resulting in more pronounced or distinct depressive symptoms during adolescence.

### 5.1. Developmental Variability

Differences in inflammatory profiles between age groups may stem from varying sources of inflammation, including psychosocial stress, infections, and obesity, which can differentially affect immune responses across the lifespan.

However, current findings in youth remain inconsistent. For example, a limited number of heterogeneous studies suggest that depression does not consistently mediate the relationship between inflammation and neurobiological dysfunction in children and adolescents [50]. Studies by Ho et al. [51,52] reported no significant correlation between IL-6, IL-1β, and TNF-α levels and depressive symptoms, although these cytokines were elevated in depressed adolescents compared to healthy controls. Similarly, Pallavi et al. [53] found increased TNF-α levels in adolescents with DD, consistent with findings in adults.

In contrast, Liu et al. [54] found, consistent with our results from the DEPOXIN project, that CRP levels in adolescents were within the normal range and showed no association with depressive symptoms. Elevated IFN-γ levels have also been reported in adolescents with depression, suggesting increased immune activation [55].

Adolescence is marked by significant neuroimmune changes that may influence cytokine levels and their relationship with depressive symptoms. Hormonal shifts, stress, and exposure to trauma may further modulate these associations differently than in adults [56].

### 5.2. Summary

In adults, elevated cytokines, e.g., IL-6, TNF-α, and CRP, are believed to contribute to depressive symptoms by affecting neurotransmitter metabolism, neuroendocrine function, and neural plasticity. In adolescents, there is evidence of heightened immune activation, though the relationship between inflammation and depressive symptoms remains inconsistent. Further research into these inflammatory pathways in youth may provide valuable insights for improving treatment outcomes for adolescent depression.

## 6. Vitamin D, Homocysteine, and Thromboxane in Depression

Both metabolites, thromboxane B2 (TXB2) and homocysteine (HCy), are related to the less discussed vitamin D in the so-called “Vitamin D-homocysteine-thromboxane axis” concerning depressive disorder.

A growing body of evidence suggests that the vitamin D–homocysteine–thromboxane axis may represent a novel integrative pathway in the pathophysiology of depression, particularly in individuals with comorbid cardiovascular, inflammatory, or metabolic disorders.

### 6.1. Vitamin D and Depression

Vitamin D functions as a neurosteroid hormone involved in neuroimmune regulation, serotonin synthesis (via modulation of tryptophan hydroxylase expression), decreased pro-inflammatory cytokines, and oxidative stress. Deficiency in vitamin D has been associated with increased depressive symptoms, especially among women [57], as well as elevated homocysteine [58] and thromboxane production [59].

This deficiency may contribute to depression by altering the availability of cholinergic, dopaminergic, and noradrenergic neurotransmitters implicated in mood regulation [60]. Additionally, vitamin D modulates inflammatory markers such as IL-6 and TNF-α, both central to the systemic inflammation observed in depression [61,62].

**Figure 5:**
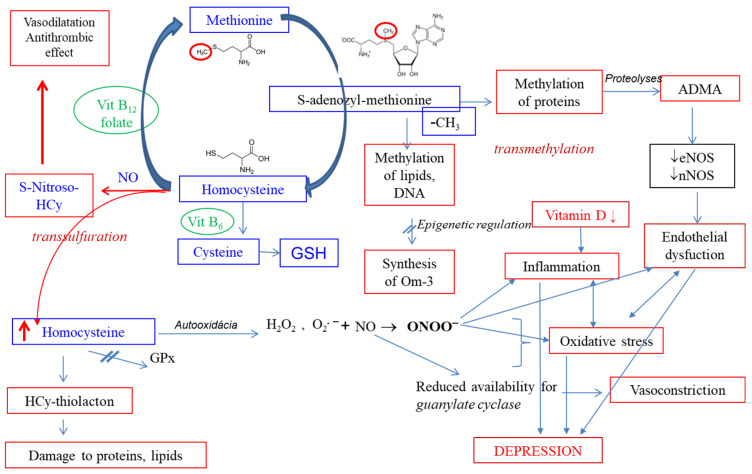
Involvement of hyperhomocysteinemia in oxidative stress and depression. ADMA—asymmetric dimethylarginine; DNA—deoxyribonucleic acid; GPx—glutathione peroxidase; GSH—reduced glutathione; HCy—homocysteine; NOS—nitroxide synthase; Om3—omega-3 fatty acids. The direction of the arrow means that one process/substance will affect another process/tissue. Modified according to [66].

Interventional studies have reported mixed findings. Kaviani et al. [63] demonstrated improvement in depressive symptoms following eight weeks of vitamin D supplementation in adults, although this did not alter platelet serotonin concentrations. In children and adolescents, supplementation with 2640 IU/day for 28 days improved depressive symptoms based on parental reports, but not self-assessments [64]. While Flanagan [65] concluded that 9 out of 11 studies support a beneficial effect of vitamin D on depression in youth, the overall evidence remains inconclusive. Notably, vitamin D status did not consistently correlate with depressive symptoms among adolescents. It remains uncertain whether supplementation is broadly beneficial or only effective among those with a deficiency (Figure 5).

The potential role of vitamin D on mental health in adults was shown, but results have been inconclusive among children. Vitamin D status did not seem to be associated with depression symptoms among adolescents. Vitamin D intake, either with supplementation or a properly balanced diet, should be included as a part of supporting mental health in children.

### 6.2. Homocysteine and Depression

Homocysteine (HCy) is a sulfur-containing amino acid involved in methylation processes and has been linked to several neurocognitive and psychiatric disorders, including depression. Its concentration is influenced by age, nutrition (particularly folate, vitamin B6, and B12 levels), and genetic polymorphisms, such as MTHFR (methylenetetrahydrofolate reductase) mutations. Elevated homocysteine contributes to inflammation, endothelial dysfunction, and increased risk of thrombosis through platelet activation (Figure 5).

In adults, elevated homocysteine has been consistently associated with increased depression risk and severity [67,68]. Gilbody et al. [69] also reported that low folate is a potential risk factor for depression, though their findings were not entirely consistent.

Evidence in pediatric populations is more limited and less conclusive. Some studies have found that adolescents with depression show elevated homocysteine and reduced folate and B12 levels compared to healthy controls. Kamath et al. [70] observed an association between homocysteine and depressive symptoms in adolescents, suggesting its potential role as a biomarker. Almeida et al. [71] found that the MTHFR C677T TT genotype, associated with impaired homocysteine metabolism, increased depression risk in youth.

However, findings from the DEPOXIN study did not reveal significant correlations between depressive symptom severity (assessed via CDI score) and either homocysteine or vitamin D levels in children and adolescents [12].

Polymorphisms in the MTHFR C677T gene (it means that at the 677 position in the gene, the expected DNA base “C” is replaced by “T”) can lead to elevated homocysteine, and it has also been studied in youth with depression [71]. Authors found that the TT genotype was associated with increased risk for depression in younger populations, likely due to its effect on homocysteine metabolism.

### 6.3. Thromboxane and Depression

Thromboxane A2 (TXA2), a pro-inflammatory eicosanoid, is rapidly converted into the stable metabolite thromboxane B2 (TXB2), which serves as a marker of platelet activation. TXB2 has been implicated in inflammatory and vascular responses and is thought to play a role in depression.

Lieb et al. [72] reported elevated TXB2 and prostaglandin E2 levels in adults with depression. Similarly, Piccirillo et al. [73] found increased TXB2 concentrations in depressed patients, which correlated positively with cortisol levels. However, no previous studies had explored thromboxane levels in pediatric depression until the DEPOXIN study.

In the DEPOXIN cohort, depressed children exhibited significantly higher TXB2 levels (411.6 ± 288 vs. 151.2 ± 89.7 pg/mL, *p* < 0.001) and lower vitamin D levels (19.0 ± 7.4 vs. 23.1 ± 5.8 ng/mL, *p* = 0.031) compared to controls [12]. TXB2 levels were positively correlated with both depression severity (CDI score: r = 0.411, *p* < 0.001) and the omega-6/omega-3 fatty acid ratio (r = 0.304, *p* = 0.03), supporting findings by DiNicolantonio and O’Keefe [74] on the role of fatty acid balance in pro-inflammatory platelet activation.

Interestingly, the correlation between TXB2 and cortisol observed in adults [73] was not replicated in the pediatric sample [14]. However, a weak, significant correlation was noted between cortisol and activation of the kynurenine pathway (KYN/TRP ratio) in adolescents (r = 0.268, *p* = 0.048) [15], suggesting an alternative stress-related pathway in this age group.

### 6.4. Summary

Collectively, these findings support the potential role of the vitamin D–homocysteine–thromboxane axis in the pathogenesis of depression, particularly in the context of inflammatory and cardiovascular comorbidities. While both adults and younger individuals with depression exhibit immune and inflammatory changes, the biological pathways involved and their clinical implications appear to vary across age groups.

## 7. Lipid Profile and Depressive Disorder

The rising incidence of depressive disorder (DD) in Western populations has been associated with substantial dietary changes over the past century. Notably, intake of omega-3 fatty acids (FAs) found in fish, grains, and some vegetables has significantly decreased, while consumption of omega-6 FAs from seed oils has markedly increased. Consequently, the dietary omega-6 to omega-3 FA ratio has shifted from approximately 1:1 to as high as 15–20:1, a trend that coincides with increased rates of depression in recent decades [75].

These developments have led to the hypothesis that omega-3 FA supplementation may be beneficial in treating depression and other mood disorders [66,76]. This is supported by the contrasting biological roles of omega-3 and omega-6 FAs: the former contributes to anti-inflammatory processes, while the latter are involved in pro-inflammatory pathways. Additionally, both influence membrane fluidity, receptor function, and gene expression related to inflammation [77].

Cholesterol, the primary lipid in the brain, is synthesized locally, mainly by astrocytes and oligodendrocytes, as it cannot cross the blood–brain barrier. In circulation, cholesterol binds to apolipoproteins to form lipoproteins. Within neuronal membranes, it influences membrane fluidity, impacting the function of membrane-bound proteins, ion channels, and synaptic transmission [78]. Despite its importance, the relationship between serum cholesterol and central nervous system function remains incompletely understood.

### 7.1. Serum Lipids and Depression

Studies investigating cholesterol levels and depression have yielded mixed results. Both low [79] and high [80,81] total cholesterol (TC) levels have been associated with depressive symptoms. Findings regarding LDL and HDL cholesterol are similarly inconsistent. Some studies report that depression is linked to decreased HDL cholesterol and apolipoprotein A, alongside increased LDL cholesterol and apolipoprotein B. However, a meta-analysis by Persons and Fiedorowicz [80] found a negative correlation between depression and LDL cholesterol.

Notably, these findings are based largely on adult populations. Age-specific patterns have also emerged: among adolescent males (12–18 years), depressive symptoms were linked to higher LDL cholesterol levels, whereas in elderly males, lower LDL levels were associated with depression [82].

In the DEPOXIN study, all participants, both patients and controls, had lipid values within normal physiological ranges [11]. To investigate the relationship between TC and depressive symptoms, participants were divided into two groups based on TC levels. No significant difference in CDI scores was found between the groups (mean CDI score: 25.7 for TC < 4.0 mmol/L, n = 36; 25.5 for TC > 4.0 mmol/L, n = 22). In agreement with Khalfan et al. [83], we found no significant differences in TC, LDL-C, HDL-C, or TAG values between patients and healthy controls. However, HDL-C levels showed a modest negative correlation with depression severity (r = −0.226, *p* = 0.043) [11].

### 7.2. Lipoprotein Subfractions and Mental Health

LDL and HDL lipoproteins are heterogeneous and can be subdivided into multiple subfractions based on size and density. LDL is typically divided into seven and HDL into ten subfractions [84,85]. Although LDL is generally viewed as pro-atherogenic and HDL as anti-atherogenic, this classification does not apply uniformly to all subfractions. Larger LDL subfractions (e.g., IDL-3, LDL-1) are considered non-atherogenic, whereas small dense LDL is strongly atherogenic. Similarly, the biological significance of intermediate HDL subfractions (I-HDL4 to I-HDL7) remains unclear, though some (e.g., I-HDL4, I-HDL5) may be non-atherogenic together with large HDL subfractions (L-HDL1 to L-HDL3), while others (e.g., I-HDL6, I-HDL7) may exhibit pro-atherogenic properties together with small HDL subfractions (S-HDL8 to S-HDL10) [86,87,88].

Although lipoprotein subfractions have primarily been studied in the context of cardiovascular disease, mental and cardiovascular disorders share overlapping pathophysiological features [66]. Research on lipoprotein subfractions in psychiatric populations is limited: to our knowledge, only two studies have investigated these in adults, and to date, the DEPOXIN study was the only one to explore them in children and adolescents [11].

Lehto et al. [89] found no association between depression and small LDL particle size, suggesting that LDL subfraction size does not mediate the link between cardiovascular risk and depression in Finnish men. In contrast, Yazici and Uçuc [90] reported elevated small dense LDL subfractions (LDL3, LDL4) in adults with depression, using the Lipoprint system (Quantimetrix Corp., Redondo Beach, CA, USA). These subfractions are recognized markers of cardiovascular risk and inflammation, supporting the hypothesis that individuals with depression may also be at increased risk of atherosclerosis.

The DEPOXIN project aimed to investigate the relationship between lipid profiles, lipoprotein subfractions, and depressive symptoms in children and adolescents [11]. Results indicated a significant inverse correlation between HDL-C levels and depressive symptom severity. Furthermore, analysis using the Lipoprint system showed that greater severity of depressive symptoms was associated with smaller HDL subfractions, whereas larger HDL subfractions were linked to symptom improvement.

### 7.3. Summary

Alterations in lipid metabolism, particularly involving HDL cholesterol and its subfractions, may be involved in the pathophysiology of depression. While total cholesterol and LDL-C levels do not appear to have a consistent relationship with depressive symptoms, reduced HDL-C and changes in HDL subfraction distribution may contribute to mood disturbances, even in younger populations. Further research is needed to clarify the role of lipid subfractions in psychiatric disorders and to explore their potential as biomarkers or therapeutic targets.

## 8. Oxidative Stress (OS)

Oxidative stress (OS) plays a dual role in human physiology. Under normal conditions, reactive oxygen species (ROS) are tightly regulated by antioxidant systems that protect cells from oxidative damage. However, when ROS production exceeds the capacity of these defenses, oxidative stress occurs, contributing to cellular dysfunction and pathology [91].

Increasing evidence supports the involvement of oxidative stress in the pathophysiology of depressive disorder. A physiological balance between pro-oxidant and antioxidant mechanisms is essential for mental health [92]. Disruption of this equilibrium has been implicated in various neuropsychiatric disorders, including depression [93]. Elevated oxidative by-products and reduced antioxidant defenses have been observed in adults with DD, indicating a pronounced oxidative imbalance [94,95].

Oxidative stress contributes to depression by promoting neuroinflammation and cellular damage. Biomarkers such as malondialdehyde (MDA), superoxide dismutase (SOD), glutathione peroxidase (GPx), reduced glutathione (GSH), thiobarbituric acid reactive substances (TBARS), and nitric oxide (NO) have been extensively studied for their role in the onset and progression of depressive symptoms. Although no single biomarker is specific to depression, combinations of several markers may aid in diagnosis and enable individualized treatment strategies.

### 8.1. Biomarker Research in Paediatric Depression

Most biomarker studies on OS in depression have focused on adults, with limited data available for children and adolescents. Results from pediatric populations often differ from those in adults due to developmental variability, differences in illness duration and chronicity, and heterogeneity in clinical presentation [96,97].

The DEPOXIN project analyzed various biomarkers in children and adolescents with depression and compared the results with healthy controls as well as an association with severity of depression and mutual associations between parameters like omega-6/omega-3 fatty acid ratio, lipid profile and HDL subfractions, secondary inflammatory markers (homocysteine, thromboxane), brain-derived neurotrophic factor (BDNF), vitamin D, oxidative stress markers, stress hormones, and tryptophan catabolism metabolites.

### 8.2. Oxidative Stress in Depression: Adults vs. Youth

Numerous studies have demonstrated increased oxidative damage and reduced antioxidant capacity in adults suffering from depressive disorder. Elevated MDA, protein carbonyls, and 8-hydroxy-2′-deoxyguanosine (8-OHdG), along with altered enzymatic activity of SOD and GPx, have been reported [98,99,100,101]. Remission is associated with normalization of these biomarkers, underscoring their potential diagnostic and therapeutic value.

In contrast, youth research has produced inconsistent results. Some studies report increased OS in depressed adolescents, while others find no significant differences. These discrepancies may reflect age-dependent variations in oxidative defense mechanisms, hormonal influences, and environmental exposures [102].

In the DEPOXIN study, depressed youth exhibited elevated 8-isoprostanes, advanced oxidation protein products (AOPP), and nitrotyrosine (NT) levels, alongside reduced GPx activity compared to healthy controls [13]. Positive correlations were observed between depression severity (CDI score) and NT, while SOD, GPx, and trolox equivalent antioxidant capacity (TEAC) were negatively correlated with the CDI score. A significantly higher serum omega-6/omega-3 fatty acid ratio was also found in patients (24.2:1) compared to controls (19.3:1) [10], with positive correlations between this ratio and both 8-isoprostanes and NT, and a negative correlation with SOD.

While catalase activity was unchanged between groups, elevated AOPP levels in patients indicate protein oxidation via alternative pathways. This contrasts with adult data suggesting deficient catalase activity as a cause of oxidative protein damage [103]. NT, a stable by-product of peroxynitrite action, was also increased in adults with depression and proposed as a biomarker, which aligns with our pediatric findings [104].

Interestingly, 8-isoprostanes and AOPP in DEPOXIN patients did not correlate with CDI scores, suggesting that they may reflect secondary pathological changes rather than primary drivers of depression. Overall antioxidant capacity (TEAC) and activities of antioxidant enzymes (SOD, CAT) did not differ significantly between groups, though inverse relationships with symptom severity suggest that oxidative imbalance plays a role in depressive pathophysiology.

### 8.3. Mechanistic Pathways Linking Oxidative Stress to Depression

OS influences multiple neurobiological pathways relevant to depression (Table 2):

- Mitochondrial dysfunction: Impaired mitochondrial activity reduces ATP production, increases ROS, and activates inflammatory cytokines (IL-1β, IL-18), which trigger the kynurenine pathway and produce neurotoxic metabolites like quinolinic acid (QA). This activates N-methyl-D-aspartate (NMDA) receptors, increases synaptic glutamate, and exacerbates oxidative damage [105,106].
- Neuroinflammation: OS promotes microglial activation, cytokine release, and IDO/TDO pathway stimulation, leading to increased ROS and neurotoxic by-products like quinolinic acid [107,108].
- Glutamate excitotoxicity: Excess glutamate induces Ca^2+^ influx, mitochondrial depolarization, and ROS production. Neuroinflammation further alters glutamate metabolism and exacerbates excitotoxicity [109,110].
- BDNF/TrkB dysfunction: OS downregulates BDNF expression via reduced CREB activity and enhanced NF-κB signaling. BDNF, in turn, supports antioxidant defenses and synaptic plasticity. Impairments in this pathway are implicated in depression and treatment resistance [105,111].
- Serotonin deficiency: OS and neuroinflammation reduce tryptophan availability by promoting IDO/TDO activity, diverting it from serotonin synthesis toward kynurenine metabolism. Pro-inflammatory cytokines also modulate the expression of serotonin transporter [112,113].
- Microbiota–gut–brain axis (MGB): OS disrupts gut microbiota, reducing tryptophan availability and impairing serotonergic and BDNF signaling [114,115]. Studies show certain probiotics can modulate serotonin-related gene expression [116].
- HPA Axis dysregulation: Chronic stress activates the HPA axis, increasing glucocorticoids and pro-oxidant signaling. Mitochondrial glucocorticoid receptors modulate oxidative metabolism, while HPA dysregulation alters gut permeability and microbiota composition [117,118].

### 8.4. Summary

The role of oxidative stress in the pathogenesis of depressive disorder is increasingly supported by research across both adult and pediatric populations. However, the heterogeneity of biomarker profiles and developmental differences in neurobiology underscore the need for age-specific investigation. While some oxidative stress markers, such as nitrotyrosine (NT), appear consistently elevated across age groups, others vary depending on hormonal, environmental, and maturational influences.

However, the discussed results of the DEPOXIN project have certain limitations.

- A small number of patients (n = 60) and healthy controls (n = 20) were enrolled in the project.
- Patients with two diagnoses were included in the project: depressive disorder (*n* = 31) or mixed anxiety and depressive disorder (*n* = 29).
- An imbalance between male (*n* = 12) and female (*n* = 48) patients. This is attributed to the higher prevalence of depressive disorder in girls and the general reluctance of boys to provide biological samples. Additionally, the patient group consisted of a maximum of 58 individuals (46 females and 12 males), while the healthy control group consisted only of 20 individuals (12 females and 8 males) for ethical reasons.
- Nutritional habits of patients and controls were not monitored, preventing a complete explanation for the baseline difference in urinary tryptophan (TRP) levels between patients and controls. However, participants were instructed to follow a standard diet with an obligation to inform the responsible doctor about any deviations in eating habits.
- Furthermore, the study did not measure the levels of quinolinic acid or kynurenic acid, which would offer a more detailed understanding of the balance in TRP metabolism (neurotoxic versus neuroprotective pathways) and the involvement of TRP metabolites in the pathophysiology of depressive disorder.
- We did not determine any of the direct markers of inflammation (IL-6, IL-1, TNF, or others) in depressed children and adolescents, for technical reasons. Patients had hsCRP levels in the physiological range. We used an indirect marker, thromboxane, to indirectly monitor the inflammatory response.

## 9. Conclusions

The DEPOXIN project has significantly advanced our understanding of the molecular basis of depression in children and adolescents. Notable findings include elevated oxidative stress markers (8-isoprostanes, AOPP, NT), reduced glutathione peroxidase (GPx) activity, and a high omega-6/omega-3 fatty acid ratio in depressed youth. Although baseline salivary cortisol levels did not differ from controls, they positively correlated with depression severity. Lipid profile analysis revealed that HDL cholesterol and the large HDL3 subfraction are inversely associated with depressive symptoms, whereas the intermediate HDL6 subfraction showed a positive correlation. From this follows that different HDL subfractions exhibit different functions. Additionally, thromboxane B2 (TXB2), a marker of platelet activation, is strongly associated with both depressive symptoms and the omega-6/omega-3 ratio.

The results of the DEPOXIN project also reveal the interrelationships between the individual systems involved in the individual chapters of the pathophysiological systems of DD.

For example, OS stimulates microglial cells and cytokine release, which activate the IDO/TDO pathway, the formation of neurotoxins (QA), and this activates the formation of ROS (Figure 4). Redirecting TRP degradation to the kynurenine pathway reduces the availability of TRP for serotonin synthesis. In addition, pro-inflammatory cytokines inhibit the expression of the serotonin transporter.

A significant modulator of systems affecting mental health, including depression, is chronic stress. Chronic stress activates the HPA axis, increases corticoid levels, stimulates pro-oxidant signaling pathways, and inhibits serotonin and BDNF synthesis (Figure 3).

These findings highlight the critical need to explore specific biomarkers in children and adolescents, pointing to a distinct redox-inflammatory phenotype in early-onset depression. Continued research is crucial to validate these markers, deepen our understanding of age-specific mechanisms, and ultimately pave the way for the development of early diagnostic tools and more precise, personalized interventions for depressive disorders throughout the lifespan.

## 10. Clinical Implications and Translational Challenges

The identification of diverse biomarkers—ranging from genetic and epigenetic signatures to neurotransmitter levels, inflammatory and oxidative stress markers, neurohormonal profiles, lipid subfractions, and neurotrophic factors—holds great promise for transforming the clinical management of depression in children and adolescents. These biomarkers could eventually support early diagnosis, stratification of patients based on biological subtypes, prognosis prediction, and personalized treatment selection. For example, specific lipid profiles or oxidative stress markers may signal a redox-inflammatory subtype of depression, guiding the use of targeted antioxidant or anti-inflammatory therapies. Similarly, neurohormonal or neurotrophic markers might inform the timing or type of pharmacological or psychotherapeutic interventions.

However, significant translational challenges remain. Most biomarker candidates lack robust validation across large, independent, and ethnically diverse pediatric cohorts. The absence of standardized protocols for sample collection, processing, and interpretation impedes reproducibility and cross-study comparability. Additionally, many biomarker assays remain costly, technically demanding, or poorly adapted for routine clinical use, particularly in child and adolescent psychiatry settings. Until these barriers are addressed through methodological harmonization, cost-effective assay development, and large-scale, longitudinal validation studies, the clinical utility of these biomarkers will remain largely theoretical. Overcoming these hurdles is essential to realizing the full potential of biomarker-guided precision psychiatry for early-onset depressive disorders.

## 11. Future Directions

Building on the insights gained from the DEPOXIN project, several avenues for future research emerge: 1. Validation of Identified Biomarkers: Replication of findings in larger, diverse, and independent cohorts is essential to establish the clinical reliability and specificity of markers such as oxidative stress indicators, salivary cortisol correlations, and HDL subfraction profiles. The patient cohort should include a larger number of enrolled boys. The diagnosis of depressive disorder should be accurately established, without other comorbidities. The treatment used should be precisely defined (monotherapy vs. combined therapy). 2. Longitudinal and Developmental Studies: Long-term studies tracking the progression of biomarker profiles from childhood through adolescence and into adulthood are needed. These will help differentiate transient vs. trait markers and illuminate the developmental trajectory of depression-related biological changes. 3. Mechanistic Exploration: Experimental studies should aim to unravel the molecular mechanisms underlying the observed associations, such as the functional role of HDL subfractions and the interplay between oxidative stress, inflammation, and lipid metabolism in paediatric depression. 4. Multi-Omics Integration: Future research should integrate genomics, epigenetics, proteomics, lipidomics, and metabolomics to capture the complexity of depression’s biological underpinnings. Such approaches may reveal novel biomarker combinations and enhance diagnostic precision. 5. Personalized Intervention Strategies: Understanding individual variability in biomarker profiles, the role of advanced neuroimaging in conjunction with biomarker research, and the development of personalized interventions based on biomarker profiles could inform personalized therapeutic strategies. This also includes targeting redox-inflammatory imbalances and metabolic dysfunctions specific to youth with depressive disorders. Given the high rate of treatment resistance (TRD) in children and adolescents with DD, it would be necessary to integrate the identified biomarkers into staging and treatment models for TRD. 6. Translational Research and Clinical Implementation: Efforts should be made to translate biomarker findings into accessible diagnostic tools or treatment monitoring systems. Salivary, blood-based, or lipid markers with strong predictive power should be explored for routine clinical use in paediatric settings.

Future investigations will facilitate the development of a new, fast-acting, and efficient treatment that will ultimately reverse depression without inducing severe side effects.

## Figures and Tables

**Table 1:** Comparative overview of neurotransmitters’ functions and presentations.

Neurotransmitter	Role in Depression	Adult Presentation	Pediatric Considerations
Serotonin	Mood, sleep, appetite regulation	Low mood, sleep disturbances	Mood swings, appetite changes
Dopamine	Motivation, pleasure, motor function	Anhedonia, psychomotor slowing	Irritability, behavioural issues
Noradrenaline	Attention, arousal, stress response	Fatigue, concentration issues	Attention deficits, heightened stress responses

**Table 2:** Pathophysiological features related to oxidative stress (data obtained from the literature).

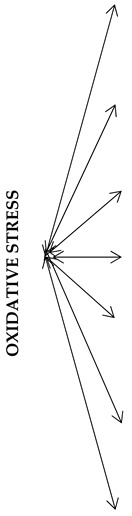	Mitochondria dysfunction	OS → ROS ↑ → ATP ↓ AP1 ↑ → pro-IC ↑ → Ca^2+^ ↑ → nNOS ↑ → Mtch dysfunction	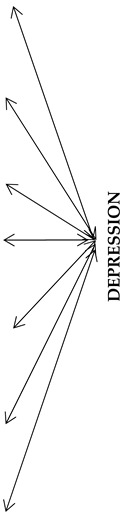
	Neuro- inflammation	OS → Mtch damage ROS ↑ → NF-κB ↑ → IDO/TDO ↑ → QA ↑ → → NMDA ↑ → GLU excitotoxicity → Ca^2+^ ↑	
	GLU excitotoxicity	OS → GLT1 ↑ OS → GLS ↑ OS → QA ↑	
	BDNT/TrkB dysfunction	OS ↑ → CREB ↓ → BDNF ↓ → NF-κB ↑	
	Serotonin deficiency	Induction of inflammation → disruption of MGB OS → IDO/TDO ↑ → KP ↑ → 5-HT ↓ OS → TNF-α ↑ → IL-1β ↑ → SERT ↓	
	MGB axis	OS → Homeostasis of neurotransmitters ↓ through BDNF/TrkB OS → 5-HT ↓	
	HPA axis dysregulation	OS → GR ↑ → negative feedback CRH/ACTH → restoration of HPA homeostasis OS → GR → NF-κB → Inflammation OS → GR agonist → IL-6/TNFα ↓ ROS ↓	

OS—oxidative stress; ROS—reactive oxygen species; ATP—adenosine triphosphate; AP1—transcription factor; pro-IC—proinflammatory cytokines; nNOS—nuclear nitroxide synthase; Mtch—mitochondrial; NFκB—nuclear factor kappa B; IDO—indoleamine 2,3-dioxygenase; TDO—tryptophan 2,3-dioxygenase; QA—quinolinic acid; NMDA—N-methyl-D-aspartic acid; GLU excitot.—glutamate excitotoxicity; GLT1—glutamate transporter 1 protein; GLS—glutaminase; TrkB—tyrosine protein kinase in brain; CREB—cAMP-response element binding protein; BDNF—brain-derived neurotrophic factor; MGB—microbiota-gut-brain axis; KP—kynurenine pathway; 5-HT—5-hydroxytryptamine (serotonin); TNF-α—tumor necrosis factor alpha; IL—interleukin; SERT—serotonin transporter; GR—glucocorticoid receptor; CRH—corticotropic hormone; ACTH—adrenocorticotropic hormone; HPA—hypothalamic-pituitary-adrenal axis. ↑means increased, ↓ means decreased, → means that system/compound influences another system/compound.

## Data Availability

Not applicable.

## Supplementary Materials

The following supporting information can be downloaded at: https://www.mdpi.com/article/10.3390/antiox14060699/s1, Table S1: Potential markers linked to depressive disorder in DEPOXIN study and other publications.
