# Supplementary material for: Current Insight into Biological Markers of Depressive Disorder in Children and Adolescents: A Narrative Review
Source: Antioxidants (Basel). 2025 Jun 9;14(6):699. doi: 10.3390/antiox14060699 (PMC12189771; doi:10.3390/antiox14060699)
Supplement: Supplementary file 1 [file antioxidants-14-00699-s001.zip › Supplement Table S1.pdf]

Table S1. Potential markers linked to depressive disorder in DEPOXIN study and other publications

Systems	DEPOXIN	Other publications	References
**Genetic and epigenetic markers**		5-HTTLPR<br>(not in adults)<br>DNA methylation<br>mtDNA methylation	17-19<br>20<br>21-23<br>24,25
**Neurotransmitters and their metabolites**	Serotonin<br>5-HIAA	Serotonin<br>5-HIAA<br>SERT<br>Noradrenalin<br>Dopamine,<br>CREB, BDNF,<br>Glutamate/GABA	27<br>26<br>31, 32<br>28<br>29<br><br>30, 33
**Endocrine markers and the Kynurenine pathway**	Cortisol<br>Kynurenine<br>Kynurenine/TRP (≈IDO)<br>5-HTP	GR<br>Hypocortisolism/<br>Hypercortisolism<br>HPA hyperactivity	34<br>37, 38<br><br>39
**Inflammation**	CRP-<br>Thromboxane	IL-6, IL-1β, TNF-α,<br>IFN-γ,<br>CRP<br>IL-10	44-49<br>54<br>44, 45, 54<br>55
**Vitamin D – Homocysteine – Thromboxane axis**	Vitamin D<br>HCy<br>TXB2	Vitamin D<br>HCy – adults<br>Folate<br>MTHFR polymorphism<br>TXB2	 67, 68<br>69<br>71<br><br>73
**Lipid profile and subfractions**	HDL-C<br>L-HDL subfractions<br>S-HDL subfractions	HDL-C, ApoA<br>LDL-C, ApoB<br>S-LDL	80-83<br><br>90
**Oxidative stress**	8-isoP, AOPP, NT<br>SOD, GPX, TEAC<br>CAT<br>Om-6/Om3 ratio	MDA, TBARS, NO,<br>Protein cyrbonyls<br>8-OHdG<br>Heterogenous results	93-95<br>98-101<br><br>102

5-HTTLPR - serotonin-transporter-linked promoter region, mt-DNA – mitochondrial DNA, 5-HIAA – hydroxyindole acetic acid, SERT – serotonin transporter, CREB - cAMP-response element binding protein, BDNF – brain-derived neurotrophic factor, GABA – gama aminobutyric acid, GR – glucocorticoid receptor, IDO – indoleamine–2,3–dioxygenase, HTP – 5–hydroxytryptophan, HPA – hypothalamic-pituitary-adrenal axis, CRP – C-reactive protein, IL – interleukin, IFN – interferon gamma, TXB – thromboxane B, HCy – homocystein, MTHFR – methylenetetrahydrofolate reductase, HDL – C – high density cholesterol, LDL – low density cholesterol, L-HDL – large HDL, S-HDL – small HDL, Apo – apolipoprotein, 8-isoP – 8-isoprostanes, AOPP – advanced oxidation protein products , NT – nitrotyrosine, SOD – supeoxide dismutase, GPx – glutathione peroxidase, TEAC – trolox equivalent antioxidant capacity, CAT – catalase, Om-6/Om-3 – omega-6/omega-3 ratio, MDA – malonedialdehyde, TBARS – thiobarbituric acid reactive substances, NO – nitrotyrosine, 8-OHdG – 8-hydroxy-2'-deoxyguanosine
